# Mouse Models Recapitulating Human Adrenocortical Tumors: What Is Lacking?

**DOI:** 10.3389/fendo.2016.00093

**Published:** 2016-07-15

**Authors:** Felicia Leccia, Marie Batisse-Lignier, Isabelle Sahut-Barnola, Pierre Val, A-Marie Lefrançois-Martinez, Antoine Martinez

**Affiliations:** ^1^UMR6293, GReD, INSERM U1103, CNRS, Clermont Université, Clermont-Ferrand, France; ^2^Endocrinology, Diabetology and Metabolic Diseases Department, Centre Hospitalier Universitaire, School of Medicine, Clermont-Ferrand, France

**Keywords:** adrenal, tumor, mouse models, PKA, WNT

## Abstract

Adrenal cortex tumors are divided into benign forms, such as primary hyperplasias and adrenocortical adenomas (ACAs), and malignant forms or adrenocortical carcinomas (ACCs). Primary hyperplasias are rare causes of adrenocorticotropin hormone-independent hypercortisolism. ACAs are the most common type of adrenal gland tumors and they are rarely “functional,” i.e., producing steroids. When functional, adenomas result in endocrine disorders, such as Cushing’s syndrome (hypercortisolism) or Conn’s syndrome (hyperaldosteronism). By contrast, ACCs are extremely rare but highly aggressive tumors that may also lead to hypersecreting syndromes. Genetic analyses of patients with sporadic or familial forms of adrenocortical tumors (ACTs) led to the identification of potentially causative genes, most of them being involved in protein kinase A (PKA), Wnt/β-catenin, and P53 signaling pathways. Development of mouse models is a crucial step to firmly establish the functional significance of candidate genes, to dissect mechanisms leading to tumors and endocrine disorders, and *in fine* to provide *in vivo* tools for therapeutic screens. In this article, we will provide an overview on the existing mouse models (xenografted and genetically engineered) of ACTs by focusing on the role of PKA and Wnt/β-catenin pathways in this context. We will discuss the advantages and limitations of models that have been developed heretofore and we will point out necessary improvements in the development of next generation mouse models of adrenal diseases.

## Introduction

Adrenocortical tumors (ACTs) are classified as benign adrenocortical adenomas (ACAs) and malignant adrenocortical carcinomas (ACCs). Most ACTs are benign, unilateral, and non-secreting adenomas, often discovered incidentally during abdominal imaging for reasons unrelated with adrenal gland (adrenal “incidentalomas”). Although less frequently, ACAs may be secreting tumors associated with endocrine hyperfunction that leads to several symptoms and significant morbidity. Indeed, clinical manifestations of secreting ACAs differ depending on their secretion profile. Cortisol-producing adenomas (CPAs) lead to Cushing’s syndrome (CS). Notably, hypercortisolism associated with unilateral ACAs is the most common form of adrenocorticotropin hormone (ACTH)-independent CS (1, 2). Aldosterone-producing adenomas (APAs) lead to primary aldosteronism (PA). APAs, together with bilateral hyperplasia, comprise 95% of all PA cases (3).

Although bilateral forms of ACTs are less frequent, several adrenal pathological conditions converge in the group of diseases termed adrenocortical hyperplasia, characterized by bilateral adrenal enlargement. Primary bilateral macronodular adrenal hyperplasia (PBMAH) is the most common and is a rare cause of CS. The report of familial forms and the bilateral nature suggest a genetic origin for PBMAH (4). Unlike PBMAH, primary pigmented nodular adrenal hyperplasia (PPNAD) is rarer but it may cause overt Cushing (5).

Contrary to ACAs, ACCs are extremely rare, with an annual incidence of 0.5–2 cases per million. However, they are highly aggressive tumors associated with poor prognosis and often diagnosed at an advanced stage (6, 7). They can occur at any age but the incidence in children is particularly high in southern Brazil due to the high prevalence of a specific TP53 mutation (8). Besides tumor growth and metastasis, clinical manifestations of ACCs are often the result of steroid hypersecretion caused by endocrine dysfunction, reminiscent of adrenal adenomas.

**Figure 1:**
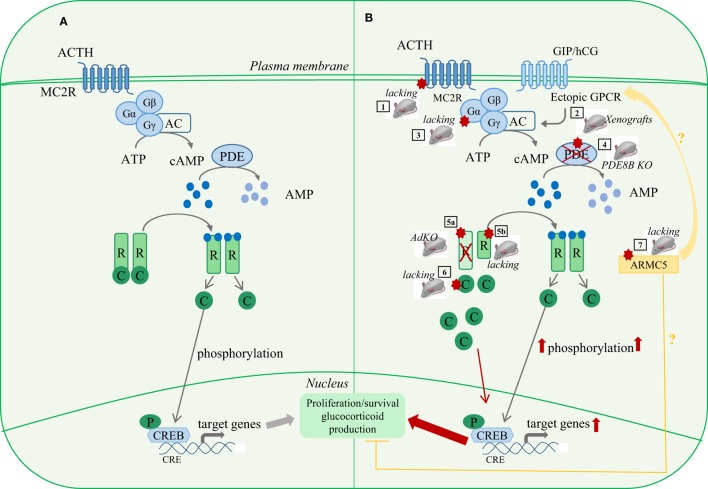
**PKA signaling pathway in adrenal tumorigenesis and related mouse models**. **(A)** Under normal conditions, ACTH binds to MC2R, a G-protein-coupled receptor (GPCR) that activates adenylate cyclase (AC) *via* Gsα, thus increasing intracellular levels of cAMP. Upon binding of cAMP to regulatory subunits (R) of PKA, the complex dissociates releasing the catalytic subunits (C) which in turn phosphorylate (among others) the cAMP-responsive element-binding protein (CREB). Once phosphorylated, CREB transcription factor regulates the expression of steroidogenic and proliferative genes. In absence of ACTH, the pathway is repressed through the activity of several phosphodiesterases (PDE) that inactivate cAMP. **(B)** Molecular alterations of several components of cAMP/PKA signaling pathway cause adrenal hyperplasias and tumors. (1) Activating mutations of *MC2R* found in PBMAH patients lead to higher activation of the pathway. No mouse model is actually available for *Mcr2* activating mutations. (2) Hormones other than ACTH (GIP/hCG/LH), through ectopic expression of several GPCRs, may induce ACTH-independent hypercortisolism in PBMAH patients. Xenograft mouse models nicely recapitulate this pathologic context. (3) Activating mutations in *GNAS* gene, encoding for the subunit α of the stimulatory G protein, causes higher activation of the pathway. These mutations are the cause of adrenal hyperplasia associated with Cushing’s syndrome in Mc-Cune Albright syndrome, whereas somatic mutations have been found in cortisol-producing adenomas. No mouse model is actually available for *Gnas*-activating mutations. (4) Inactivating mutations in PDE genes lead to an accumulation of cAMP, thus causing the persistent activation of the pathway in absence of ACTH and they have been associated with PBMAH and hypercortisolism. Whole-body *PDE8B* knockout mice developed mild hyperplasia. (5a) Most inactivating mutations in the *PRKARIA* gene, encoding the 1α regulatory subunit (R1α) of the PKA, lead to aberrant transcripts and to the absence of the protein, resulting in constitutive activation of PKA. Adrenocortical-specific *Prkar1a* knockout (AdKO) mice develop a PPNAD-like syndrome with adrenal hyperplasia and ACTH-independent hypercorticosteronism. (5b) Some *PRKARIA* gene mutations lead to expressed mutated (truncated) R1α proteins that also cause increased PKA activation. These mutations are associated with a more severe phenotype for reasons still not understood to date. No mouse model is available for expressed mutated R1α proteins. (6) Mutations in the *PRKACA* gene encoding for the catalytic subunit α (Cα) of the PKA alter its interaction with regulatory subunits leading to constitutive activation of PKA-Cα and increased steroidogenesis. These mutations have been identified in many patients with cortisol-producing adenomas. No mouse model is actually available for *Prkaca* activating mutations. (7) Inactivating germline and somatic mutations in the armadillo repeat-containing 5 (*ARMC5*) gene have been identified in ~50% of patients with PBMAH. The function and the mechanisms by which ARMC5 contributes to the pathogenesis of PBMAH are unknown. *In vitro* studies suggest a role in steroidogenesis and apoptosis processes and a possible interplay with the PKA pathway, which is supported by the association of *ARMC5* mutations with particular expression profile of GPCRs. No loss of function mouse model is actually available for *Armc5*.

**Figure 2:**
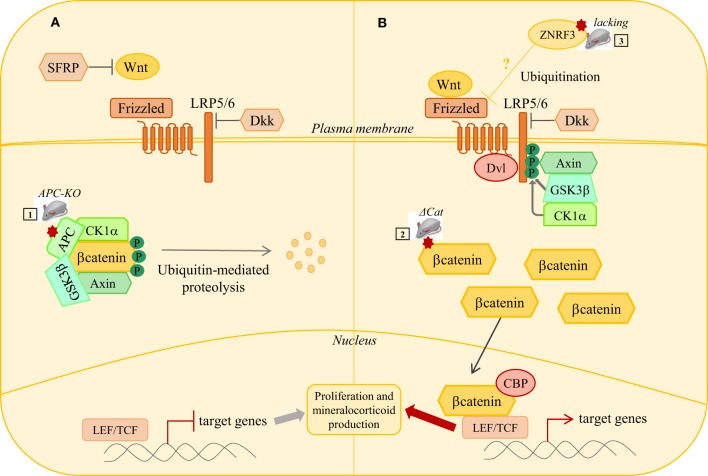
**WNT/β-catenin signaling pathway in adrenal tumorigenesis**. **(A)** In the absence of Wnt ligands, b-catenin is phosphorylated by a complex composed of GSK3b, CK1, APC, and AXIN. This phosphorylation targets b-catenin to proteosomal degradation, thus preventing its nuclear translocation. In this context, the T cell-specific factors (Tcf)/lymphoid enhancer-binding factor (Lef) represses target genes expression through association with transcriptional inhibitors. Secreted frizzled related proteins (SFRP) can inhibit extracellular Wnt signaling. **(B)** When WNT signaling is active, binding of Wnt ligands to their receptor complex (Frizzled/LRP5/6) induces the recruitment of Disheveled (Dsv) to the cytoplasmic domain of Frizzled and the phosphorylation of the cytoplasmic tail of LRP5/6 by CK1 and GSK3b. This induces delocalization of Axin to LRP5/6 and sequestration of the degradation complex. b-catenin degradation is, thus, reduced and it accumulates. Accumulated b-catenin enters the nucleus, binds to LEF/TCF transcription factors, and activates transcription of target genes. Mutations in several members of WNT/β-catenin signaling pathway have been identified in adrenal hyperplasias, adenomas, and carcinomas. Several mouse models have been developed to induce constitutive β-catenin activation either through adrenal cortex specific loss of *Apc* (1) or the expression of a protein resistant to phosphorylation and ubiquitin-dependent degradation (2). Loss of ZNRF3, a potential Wnt signaling inhibitor, has been identified as the major genetic alteration in adrenal carcinoma. Mouse model with adrenal specific inactivation of *Znrf3* is required to assess its causal involvement in ACC.

Over the last 5 years, genetic analyses of patients with sporadic or familial forms of ACTs has resulted in identification of alterations in a new set of genes, most of them being involved in cAMP/protein kinase A (PKA) and Wnt/β-catenin signaling pathways (9–11) (Figures 1 and 2). The major difference in the prevalence of ACA and ACC in patients suggests that adenomas are not precursors of malignant neoplasms. Moreover, the malignant transformation of a benign and non-functional adrenal tumor is very rare (12–14). Notably, the risk that an adrenal incidentaloma progresses to a malignant tumor has recently been estimated as almost zero by the *European Society of Endocrinology* (ESE). Consequently, European recommendations for the clinical management of patients with non-functional ACAs have been reconsidered and modified to avoid unnecessary procedures (European Congress of Endocrinology, May 2016, Munich, Germany – Symposium 5: ESE clinical guidelines: Management of adrenal incidentaloma: http://www.ece2016.org/scientific-programme/). By contrast, secreting ACAs are surgically removed, which prevents evaluation of a possible benign to malignant continuum in functional adenomas. In fact, the hypothesis that ACC could develop in a multistep process from normal adrenal to adenoma followed by malignant transformation relied on one case report in which a carcinoma emerged in the center of a surrounding benign ACT (15). However, some genome-wide approaches performed on independent cohorts to analyze genomic changes and gene regulation in ACTs suggests that cancers could result from pangenomic cumulative changes occurring in a multistep tumor progression (16–18). Although it is important to predict to what extent a benign lesion can be considered as the precursor of malignancy, analysis of patients’ data may not be sufficient to provide a definitive conclusion.

The use of small animals for modeling tumors in a controlled experimental manner is a valuable strategy to explore the functional significance of mutations, to dissect mechanisms underlying both adrenocortical tumorigenesis and endocrine disorders, and to provide *in vivo* tools to screen for novel therapeutic approaches. To date, several genetically modified and xenografted mouse models have been developed to investigate the involvement of specific pathways and the heterogeneous nature of ACTs, respectively. Although models established until now have shed light on important aspects of adrenocortical diseases, many of them failed to fully mimic tumors found in human adrenals (Table 1). Hence, there is a strong need to develop relevant mouse models to shed light on mechanisms involved in the initiation and progression of adrenal tumors. In this article, we provide an overview of the existing mouse models (xenografted and genetically engineered) of ACTs relevant to human ACTs, including adrenal hyperplasia. We will discuss limitations of models that have been developed heretofore and we will point out necessary improvements in the development of next-generation mouse models of adrenal diseases. Notably, these models should allow, on the one hand, to firmly establish the role of newly identified genes in adrenocortical tumorigenesis and, on the other hand, to explore the interplay between pathways shown to be associated with ACTs (e.g., cAMP/PKA and Wnt/β-catenin).

**Table 1:** **Current mouse models of adrenocortical tumors and their limitations**.

Model	Gene	Promoter/driver	Adrenocortical phenotype	Limitation	Reference
Men1^±^	*Men1*	Whole-body KO	Hyperplasia, adenoma, carcinoma (lack of tumor grade definition)	Multiple tumors	(19, 20)
Prkar1a^2Δ/+^	*Prkar1a*	*EIIA-Cre*	No adrenal phenotype	No adrenal phenotype	(21)
tTA/X2AS	*Prkar1a*	Tet-Off system	Hyperplasia maintaining of X-zone	Technical limitations, reproducibility	(22)
AdKO	*Prkar1a*	*0.5 Akr1b7-Cre*	Expansion of X-like zone with *zona fasciculata* features, autonomous corticosterone secretion	Late phenotype	(23)
Pde8b−/−	*Pde8b*	Whole-body KO	Mild hyperplasia	No adrenal-specific ablation	(24)
ΔCat	*Ctnnb1*	*0.5 Akr1b7-Cre*	Hyperplasia, adenoma and ectopic *zona glomerulosa*, hyperaldosteronism	Rare carcinomas	(25)
APC-KO	*Apc*	*Sf1-Cre^low^*	Hyperplasia progressed to microscopic and macroscopic adenomas	Progression to carcinoma was never observed	(26)
APC KO-H19^ΔDMD^	*Apc Igf2/H19 I*CR	*Sf1-Cre^low^*	Hyperplasia and adenomas, more severe phenotype than APC-KO mice	One carcinoma	(26)
PEPCK-IGF	*Igf2*	*Pepck-Igf2*	Hyperplasia	No adrenocortical tumors	(27)
ΔCat; AdIgf2	*Ctnnb1 Igf2*	*0.5 Akr1b7-Cre*	Hyperplasia, adenoma, slight increased proliferation compared to ΔCat mice	Moderate effect on tumor progression	(28)
		*0.5 Akr1b7-Igf2*			
		*4.5 Scc-Igf2*			
Acd^acd/acd^; p53^±^	*Acd, Tp53*		Increased development of ACC compared to Acd^acd/acd^	Multiple tumors	(29)
Inhα/TAg	*SV40 (large T antigen)*	*6kb inhibin*α*-TAg*	Malignant ACTs developing upon gonadectomy	Unrelated to human pathology	(30)
AdTAg	*SV40 (large T antigen)*	*0.5 Akr1b7-TAg*	Rapidly evolving tumors		(31)
YAC TR	*NR5A1*	YAC transgene	Hyperplasia and tumors	Tumors with gonadal phenotype	(32)

*MEN1, multiple endocrine neoplasia type 1; Prkar1a, Protein kinase cAMP-dependent regulatory subunit type I alpha; Akr1b7, aldo-keto reductase family 1, member b7; Pde8b, phosphodiesterase 8b; Ctnnb1, catenin (cadherin-associated protein), beta 1; APC, adenomatous polyposis coli; KO, knockout; ICR, imprinting control region; PEPCK, phosphoenolpyruvate carboxykinase; IGF2, insulin-like growth factor 2; Acd, adrenocortical dysplasia; TP53, tumor protein P53; TAg, tumor antigen; SV40, simian virus 40; YAC, yeast artificial chromosome; Nr5a1, nuclear receptor subfamily; tTA/X2AS, transgenic mouse carrying an antisense transgene for Prkar1a exon 2 (X2AS) under the control of a tetracycline responsive promoter; ΔCat, Catnb^lox(ex3)^ line x 0.5AkR1b7-Cre*.

## Benign Adrenocortical Tumors and Associated Hypersecretion Syndromes

### Primary Bilateral Macronodular Adrenal Hyperplasia

Primary bilateral macronodular adrenal hyperplasia is a rare cause of CS, accounting for <2% of all endogenous CS cases (33, 34). PBMAH is characterized by the presence of adrenocortical nodules larger than 10 mm and it is often diagnosed in patients between 40 and 60 years of age, with clinical signs of cortisol excess and suppressed levels of plasma ACTH. PBMAH was first described by Kirschner and colleagues (35) and it was named ACTH-independent macronodular adrenal hyperplasia (AIMAH). However, it has been recently found that cortisol production in PBMAH is not truly ACTH independent, since a population of adrenocortical cells in the hyperplastic tissue can produce ACTH that in turn stimulates cortisol secretion through autocrine and paracrine mechanisms (36). Hence, the term “ACTH independent” is not entirely appropriate for this disorder. Although in the majority of cases, PBMAH appears to be sporadic, several cases of familial clustering have been reported in the last years (37–39). However, the true prevalence of the familial vs. sporadic form is unknown, as systematic familial screening has not been conducted.

#### Aberrant Hormone Receptors in PBMAH and Related Mouse Models

The mechanism by which cortisol production is stimulated in PBMAH, despite suppressed plasma ACTH, was previously unknown and was referred to as being “autonomous.” Several groups have then shed light on the pathogenesis of hypercortisolism in PBMAH. They reported that in most patients with PBMAH and in some adenomas, cortisol secretion is regulated by hormones other than ACTH, through the aberrant expression of several G-protein-coupled receptors (GPCRs) that are normally absent (ectopic) or expressed at lower levels in the adrenal cortex (40–42). The GPCRs for gastric inhibitory polypeptide (GIP), catecholamines, vasopressin, serotonin, and luteinizing hormone/human chorionic gonadotropin (LH/hCG) have been shown to be involved in adrenal CS development (42). GIP-dependent CS has been reported in patients with PBMAH and with unilateral adenomas (33, 43, 44). In these patients, plasma cortisol levels were increased following meals, despite ACTH suppression, and paralleled postprandial elevation of GIP plasma concentrations. Hypercortisolism associated with aberrant LH/hCG receptors was first identified in a woman with transient CS during sequential pregnancies and persistent CS after menopause (45). Since then, several cases of PBMAH with aberrant LHR have been reported, alone or in association with GIPR (45, 46). The molecular mechanisms responsible for aberrant expression of these receptors are unknown as no genetic mutations have been found in the coding or regulatory regions of receptor genes. Furthermore, whether expression of these receptors is a primary or a secondary event in the pathogenesis of PBMAH is still a matter of debate. Several lines of evidence support the hypothesis that it is a causative factor. First, aberrant receptors are almost universally present in PBMAH and at early stages (47). Second, the same aberrant receptors were found in all members of some affected families (37, 48), although this was not found in a known Brazilian family (39). Further data supporting a triggering role of ectopic receptors in PBMAH came from xenotransplantation mouse models. Indeed, to investigate the role of ectopic GIPR and LHR expression in the development of PBMAH, Mazzuco and colleagues used an *in vivo* model of cell transplantation and tissue reconstruction. Primary bovine adrenocortical cells were genetically engineered to express GIPR or LHR (retrovirus-mediated enforced expression) and transplanted under the kidney capsule of adrenalectomized immunodeficient mice (49, 50). Transplantation of GIPR- or LHR-expressing cells induced the formation of hyperplasic and hypertrophic adrenocortical tissues. The growth advantage provided by aberrant receptors expression at least relied on increased proliferation rates of transplants but the downstream mechanisms supporting proliferation were not explored. These models nicely recapitulated the context of human PBMAH with GPCR-dependent CS. Indeed, LHR-dependent CS is not exclusively observed during pregnancies or after menopause (41). Likewise, in the LHR xenotransplanted model, the hyperplastic tissue formed in the absence of supraphysiological levels of plasma LH. This shows a direct role of aberrant LHR expression in the pathogenesis of PBMAH with LH-responsive CS, even though the molecular mechanisms leading to ectopic expression of GPCRs in adrenocortical cells are still unknown.

Xenotransplantation models developed by Mazzuco et al. are not easy to manage for long-term follow-up or for assessment of therapeutic strategies, because immunodeficient mice with CS have short life expectancy. However, this elegant approach combining genetic engineering and cell transplantation of bovine adrenal cells in mice may be a useful tool to test the cooperation of multiple genetic alterations in the tumorigenic process (51) or when genetic alterations may be not relevant in mouse (e.g., *KCNJ5* gene, see Aldosterone-Producing Adenomas: WNT Pathway, KCNJ5, and Lack of Mouse Models).

#### Familial Forms of PBMAH, Genetic Alterations, and Related Mouse Models

Reports of rare familial forms and the bilateral nature of these tumors support a genetic origin of PBMAH. Many genes are associated with the development of PBMAH, including genes causing hereditary familial tumor syndromes, such as *APC* (52, 53), *MEN1* (54), and *FH* (55). Moreover, several reports pointed out genes involved in the cAMP/PKA signaling pathway, such as *PDE8B* and *PDE11A* (56, 57), *MC2R* (58), *GNAS* (59), and *PRKACA* (9) (Figure 1). Mutations in members of cAMP/PKA pathway are predicted to over-activate the pathway but they have been observed in a limited number of patients (Figure 1).

Germline inactivating mutations of the *MEN1* gene cause a complex genetic syndrome named multiple endocrine neoplasia type 1 (MEN1) characterized by endocrine and non-endocrine tumors (60). PBMAH occurs in ~21% of MEN1 patients (54). Two whole-body *Men1* KO mouse models have been reported. They nicely recapitulate the spectrum of tumors of MEN1 syndrome, including adrenocortical lesions. Specifically, whereas homozygous whole-body *Men1* KO is embryonic lethal (19, 20), heterozygous mice are viable and develop tumors similar to those found in the human disease, including adrenocortical hyperplasia that seems to progress from adenoma to carcinoma (20). Of note, characterization of the histological phenotype, allowing establishment of tumor grade, was not detailed in this paper. Therefore, the conclusion that a multi-step tumor progression process occurs in *Men1* adrenal tumors should be taken with caution (Table 1). Since *MEN1* has recently been identified as a significantly mutated gene in ACC (11), *Men1* KO mice could be useful to identify novel actors and mechanisms underlying the evolution of benign ACTs to malignancy.

Although the bilateral nature and the multifocal nodules suggest an important role of genetic factors in PBMAH, genetic defects summarized above account for only a few cases of this adrenal disease. More recently, inactivating germline and somatic mutations in the armadillo repeat-containing 5 (*ARMC5*) gene have been identified in ~50% of patients with apparently sporadic PBMAH and also in a large family with genetically transmitted PBMAH (10, 39, 61, 62). *ARMC5* is a tumor suppressor gene with the typical “two-hit” pattern of mutations: a first germline mutation and a second somatic one. The function and the mechanisms by which ARMC5 contributes to the pathogenesis of PBMAH are unknown. However, *in vitro* studies suggest a role in steroidogenesis and apoptosis processes (10). Indeed, *ARMC5* inactivation in cultured adrenocortical cells decreases the expression of *MC2R* and of various steroidogenic enzymes, both in basal conditions and after cAMP stimulation (10), suggesting that it may interfere with PKA pathway by impairing the stimulation of its target genes (Figure 1). Increased cell survival upon ARMC5 inactivation is proposed to trigger hyperplasia while subclinical CS could be the result of the major increase in adrenal mass that would compensate for decreased per-cell steroidogenic activity. Furthermore, *ARMC5* mutations seem to be associated with particular expression profile of GPCRs, i.e., beta-adrenergic and dopamine receptors (10). This observation supports the hypothesis of a link between ARMC5 and PKA signaling, as the abnormal expression of GPCRs leads to activation of PKA signaling, normally triggered by the ACTH receptor (MC2R) (Figure 1). Further *in vitro* studies and the development of knockout models are required to shed light on the ARMC5-dependent network that triggers development of PBMAH and CS. Notably, the combination of adrenal targeted GPCRs overexpression and *Armc5* knockout could provide information on a possible cooperation between ARMC5 and PKA signaling.

### Primary Pigmented Nodular Adrenocortical Disease and Related Mouse Models

**Figure 3:**
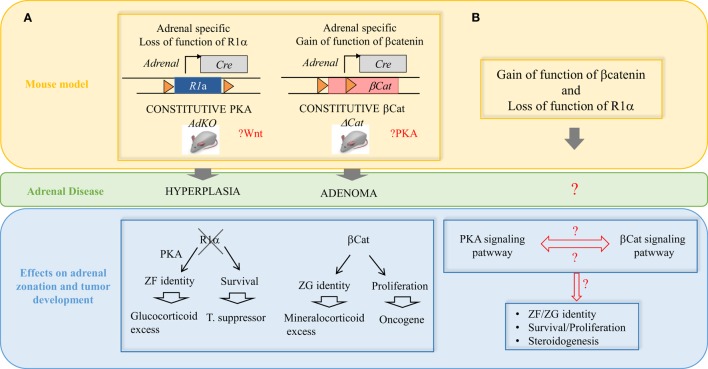
**Interplay between the WNT/β-catenin and the cAMP/PKA pathways in the pathogenesis of adrenal hyperplasias and tumors**. **(A)** Two mouse models recapitulating some of the most frequent alterations found in adrenal tumors in patients. AdKO mice developed cortical hyperplasia as a result of constitutive PKA activation due to gene inactivation of R1a regulatory subunit of PKA. The increased PKA signaling also favored *zona fasciculata* cell identity and glucocorticoid excess. ΔCat mice developed cortical adenoma as a result of constitutive b-catenin due to deletion of exon 3 in the gene encoding the b-catenin (*Ctnnb1)* leading to protein stabilization. Increased b-catenin activation also induced ectopic differentiation of *zona glomerulosa* and aldosterone excess. **(B)** Our models demonstrated that *Prkar1a* is a tumor suppressor and *Ctnnb1* is an adrenal oncogene but secondary genetic alterations are required for malignant progression. A possible interplay between the WNT/β-catenin and the cAMP/PKA pathways in the adrenal cortex zonation and tumorigenesis is an important point to further investigate. The question can be genetically addressed by using compound transgenic mice based on previous available models and carrying both β-catenin and PKA constitutive activation.

Primary pigmented nodular adrenocortical disease is a type of adrenal hyperplasia characterized by the presence of cortisol-secreting bilateral adrenal micronodules (<1 cm). PPNAD is the most common endocrine manifestation of Carney complex disease (CNC), an autosomal-dominant multiple neoplasia syndrome (63). More than 60% of CNC patients harbor mutations in the *PRKARIA* gene, encoding the regulatory subunit 1α (R1α) of PKA (64) (Figure 1). Specifically, inactivating heterozygous germline mutations are observed in about two-thirds of Carney Complex patients and loss of heterozygosity (LOH) has been reported, indicating that *PRKARIA* acts as a tumor suppressor gene (64). Interestingly, somatic inactivating mutations have also been found in ACTs (65). Several mouse models have been developed to target *Prkar1a* gene inactivation by different strategies (Table 1). Total *Prkar1a* KO mice had severe defects in mesoderm development and died at E9.5 (66). Mice heterozygous for a null allele of *Prkar1a* developed several tumors but had no adrenal phenotype (21). An antisense RNA approach was developed to achieve a more severe loss of R1α than in heterozygous KO mice (22). This allowed a 70% decrease in *Prkar1a*, causing several neoplastic manifestations, including mild adrenocortical hyperplasia. Interestingly, adrenocortical manifestations in the antisense RNA model included maintenance of the X-zone, a normally transient zone of fetal origin. To achieve bi-allelic inactivation without compromising mouse survival, we generated an *ad*renocortical-specific *Prkar1a k*n*o*ckout mouse model (AdKO) (23) by crossing *Prkar1a*-floxed strain with *Akr1b7*-Cre mice, thereby targeting specific genetic ablation in adrenocortical cells from E14.5 (67) (Figures 1 and 3; Table 1). These mice developed a PPNAD-like syndrome with ACTH-independent hyper-corticosteronism and adrenal hyperplasia, composed of hypertrophic cells emerging from the innermost cortex. Endocrine overactivity was associated with unbuffered PKA catalytic activity, which resulted in overexpression of steroidogenic genes. Interestingly, fetal-like hyperplasia centrifugally expanded with aging at the expense of the normal adult cortex, which underwent progressive atrophy. This mouse model confirmed the important role of PKA pathway in adrenal hyperplasia and shed light on possible mechanisms responsible for PPNAD. The most likely mechanism to explain the defective zonal differentiation and cell renewal in AdKO mice is an impaired capacity of adult progenitor cells of the outer cortex to undergo centripetal differentiation and increased survival of cells in the inner cortex that would then cumulate both fetal-like and fasciculata cell features. We further showed that PKA-dependent induction of mTOR signaling was one of the mechanisms participating in the resistance to apoptosis, leading to both hyperplasia and cell hypertrophy, typical of PPNAD (68, 69). Altogether, these observations suggest that PPNAD should be considered as a developmental disease.

To further elucidate the molecular mechanisms by which PKA pathway contributes to the initiation and/or development of adrenal disorders, it is essential to provide deeper insight of its interplay with other signaling pathways (Figure 3). Interestingly, WNT pathway activation has been involved in both PBMAH and PPNAD (70, 71) (Figure 2). Furthermore, as we will discuss in next sections, WNT pathway is also associated with ACA and ACC. A possible interplay between WNT/β-catenin and cAMP/PKA pathways in the pathogenesis of adrenal hyperplasia and tumors would be an important point to further investigate with mouse models (Figure 3).

### Cortisol-Producing Adenomas: PKA Pathway, PRKACA Mutations, and Lack of Mouse Models

As discussed above (Sections “Familial Forms of PBMAH, Genetic Alterations, and Related Mouse Models” and “Primary Pigmented Nodular Adrenocortical Disease and Related Mouse Models”), a number of genetic defects in the cAMP/PKA pathway have been associated with adrenal hyperplasia and related to cortisol hypersecretion (Figure 1). Somatic mutations of *GNAS* (72–74) and *PRKAR1A* (75) have also been found in CPAs (Figure 1). However, these mutations only accounted for a small subset of CPAs, which represent a relevant cause of CS. Recently, Beuschlein and collaborators identified a hotpsot mutation (L205R) in the *PRKACA* gene, encoding the catalytic subunit α (Cα) of PKA, in more than one-third of patients with CPA (9). Four other groups subsequently reported the same mutation (72–74, 76). The *PRKACA* L205R mutation results in constitutive PKA activation (9, 74), which is a very likely cause of ACT formation (Figure 1). Supporting this hypothesis, patients with somatic mutations had adenomas, whereas patients with germline duplications had bilateral hyperplasias. Hence, *PRKACA* mutations, together with the previously identified *GNAS* and *PRKAR1A* inactivating mutations, strongly support a crucial role of cAMP/PKA pathway in the tumorigenesis of CPAs. The development of knock-in mouse models bearing *PRKACA* activating L205R mutation is required to evaluate its driver potential and to provide new insights into the mechanisms underlying PKA-dependent tumorigenesis in the context of cortisol-producing ACAs. In addition, adding extra copies of *Prkaca* in mouse, by additive transgenesis or targeted transgenesis at the *Rosa26* locus, would provide an opportunity to explore the pathogenic, maybe oncogenic, potential of PKA signaling. Such complementary mouse models would help understanding if gain-of-function mutations and gain of copy number alterations result in distinct adrenal lesions. Activating mutations in *CTNNB1*, the gene encoding β-catenin, have also been identified in CPAs (77). Interestingly, the recently identified *PRKACA* mutations were shown to be mutually exclusive with *CTNNB1* mutations (72–74, 76). Therefore, a possible interplay between WNT/β-catenin and cAMP/PKA pathways in CPAs remains to be investigated. Once again, mouse models should be invaluable tools to address this question *in vivo* (Figure 3).

### Aldosterone-Producing Adenomas: WNT Pathway, KCNJ5, and Lack of Mouse Models

In recent years, high throughput next-generation sequencing technologies have allowed major advances in the knowledge of the genetic bases of APAs. By comparing the APA exome to the germline exome, recurrent somatic mutations have been identified in genes coding for ion channels and transporters regulating the cell membrane potential. Specifically, mutated genes encoding ion channels include *KCNJ5*, which encodes the G-protein activated potassium channel GIRK4, and is mutated in about 26–40% of APAs (78, 79) and *CACNA1D* and *CACNA1H* genes encoding for voltage-dependent calcium channels (80, 81). Genes encoding regulators of the cell membrane potential include two ATPases, *ATP1A1* and *ATP2B3* (82). All these mutations ultimately lead to increased intracellular calcium and abnormal activation of calcium–calmodulin-dependent kinase (Ca^2+^–CAMK) signaling, which plays a central role in aldosterone production. Before identification of these novel APA-associated genes, several mouse models of potassium channels inactivation (*KCNK3/KCNK9* inactivation models) allowed understanding the effects of calcium homeostasis disruption and of some important aspects of PA (83–85). However, none of these models recapitulated hyperplasia and/or tumor development observed in human disease. Although one cannot exclude that mouse adrenal context may not be relevant to reproduce pathophysiological conditions associated with APA formation in humans, this suggests that in APAs, the tumorigenic potential could rely on a yet unidentified alteration. Among the newly identified genes, *KCNJ5* mutations represent the most frequent genetic defects in APAs, with higher prevalence in the Japanese population (79). Thus, animal models of *KCNJ5* inactivation are warranted to confirm a central role for this gene in the initiation of APAs. However, *Kcnj5* mRNA and KCNJ5 protein are not expressed in the rat adrenal cortex, suggesting that it does not play a role in adrenal steroid production in this species and very likely in mice (86). Therefore, the demonstration that loss of *KCNJ5* is sufficient to initiate both hyperaldosteronism and tumor development will require genetic approaches in non-mouse systems, such as primary bovine adrenocortical cells and tissue reconstruction in xenografted mice.

Both cAMP/PKA and WNT/β-catenin pathways have been involved in the development of CPAs. Similarly, WNT/β-catenin pathway plays an important role in APA development, in addition to calcium signaling. Indeed, by generating a mouse model with constitutive β-catenin activation in the adrenal gland (ΔCat model), we found that these mice developed progressive dysplasia and hyperplasia, ectopic differentiation of *zona glomerulosa* (ZG), and increased aldosterone production (Table 1). The ΔCat model will be further discussed in the ACC section. In a subsequent paper, Berthon and colleagues reported that WNT/β-catenin pathway was aberrantly activated in 70% of a series of 47 patients, which was the most frequent alteration reported in APAs (87). Furthermore, a recent study has reported *CTNNB1* mutations in 5.1% of a cohort of 198 APAs (88). These mutations were associated with stabilized β-catenin, suggesting activation of WNT pathway (Figure 2). Because of the higher frequency of WNT/β-catenin activation than of *CTNNB1* mutations, it is essential to expand our knowledge of the other causes of aberrant WNT/β-catenin activation, by investigating other members of the pathway or possible crosstalks with other pathways. Interestingly, decreased expression of the WNT inhibitor *SRFP2* was shown to contribute to deregulation of WNT/β-catenin pathway in the adrenal (87) (Figure 2).

## Adrenocortical Carcinomas: The Lack of Mouse Models

Adrenocortical carcinomas are extremely rare, with an annual incidence of 0.5–2 cases per million in adults. However, they are highly aggressive tumors associated with poor prognosis and often diagnosed at an advanced stage for which available treatments are rarely curative. The overall 5-year survival rates range from 10 to 40% (6, 7). ACCs can occur at any age, but the incidence in children is particularly high in southern Brazil due to the high prevalence of a specific germline mutation (p.R337H) of the *TP53* tumor suppressor gene (8). Pediatric and adult ACCs differ in genetics and many other ways (89, 90), as will be further discussed in Section “Pediatric ACCs: TP53, SF1, and Related Mouse Models.” Although most ACCs arise sporadically, an increased incidence of ACCs has been reported in some genetic syndromes, such as familial adenomatous polyposis (FAP), characterized by mutations in *APC* and elevated Wnt/β-catenin signaling, and Beckwith–Wiedemann syndrome (BWS), characterized by elevated expression of insulin-like growth factor-2 (*IGF2*) (91, 92).

The developmental context of the disease is crucial for the treatment and management of adrenal tumors. Therefore, there is a strong need to generate mouse models resembling human pathology, to identify the mechanisms involved in benign to malignant progression. Up to now, the three most frequent alterations that have been reported in ACC patients include overexpression of IGF2 (93, 94), activation of WNT/β-catenin signaling pathway (11, 77, 95), and inactivation of TP53/RB pathway (96, 97). Several mouse models of deregulation of these pathways have been generated, which allowed better understanding of adrenal tumorigenesis. However, none of these models was able to recapitulate full-fledged ACC development (Table 1). In the following sections, we summarize mouse models of ACC generated up to now, and focus on what is still lacking for a better understanding of adrenal tumor initiation and progression.

### Mouse Models of WNT/β-Catenin and IGF2 Signaling Activation Are Insufficient to Trigger ACC Formation

The WNT/β-catenin pathway is essential for embryonic development and cell renewal in adult adrenal cortex where β-catenin is expressed and active in ZG (98) (Figure 3). Several tumor profiling studies have reported mutations in β-catenin gene (*CTNNB1*) in both ACAs and ACCs (77, 99, 100), suggesting that β-catenin activating mutations could be involved in adrenal tumor initiation and progression to malignancy. To assess this hypothesis, we have generated a mouse model of constitutive β-catenin activation in the adrenal cortex (ΔCat mice) (25) (Figures 2 and 3; Table 1). ΔCat mice were generated by mating mice harboring a floxed allele of β-catenin [*Catnb^*lox(ex3)*^*] (101) with mice expressing the Cre recombinase in steroidogenic cells of the adrenal cortex, through the *Akr1b7* promoter region (67). Cre-mediated excision of the third exon of *Ctnnb1* gene prevents β-catenin phosphorylation and ubiquitin-dependent degradation, which induces accumulation of the protein and constitutive activation of its target genes. ΔCat mice showed adrenal hyperplasia and ectopic differentiation of ZG. However, aggressive tumor formation was only observed in a subset of 17-month-old animals. Our model demonstrated that *Ctnnb1* was an adrenal oncogene but it also suggested that secondary genetic alterations were required for malignant progression. Through a different approach, Hammer and colleagues obtained similar results by generating mice with adrenal cortex specific loss of *Apc* (APC KO mice) (26), a component of the multi-protein destruction complex of β-catenin, to target WNT pathway activation (Figure 2). As ΔCat mice, APC KO mice displayed hyperplasia. Furthermore, hyperplasia progressed to microscopic and macroscopic adenomas as early as 15 weeks of age, but progression to carcinoma was never observed (Table 1). Results from our two groups suggest that constitutive activation of β-catenin initiates benign tumor development but is not sufficient to trigger malignant evolution.

Insulin-like growth factor-2 is a growth factor involved in the control of cell proliferation and inhibition of apoptosis and it was hypothesized to interact with the Wnt/β-catenin pathway. In sporadic adrenal tumors, IGF2 is overexpressed in 80–90% of ACCs but not in ACAs (93, 94). Several transgenic mouse models have been generated to explore the role of IGF2 in adrenal tumorigenesis (Table 1). Mice with overexpression of IGF2 under the control of phosphoenolpyruvate carboxykinase (*PEPCK*) promoter showed four- to sixfold elevation of serum IGF2 levels and mild adrenocortical hyperplasia but did not develop ACTs (27), suggesting that IGF2 overexpression was not involved in initiation of adrenal tumorigenesis. We have generated transgenic mice with *ad*renal cortex specific overexpression of *IGF2* (AdIgf2 mice) (28) (Table 1). These mice had up to sevenfold higher basal levels of IGF2 (up to 87-fold higher with ACTH stimulation), but again they did not show tumor formation despite a mild increase in cortical cell proliferation. These results indicated that IGF2 alone could stimulate adrenal cortex proliferation but it was not able to induce oncogenic transformation. The mitogenic effect of IGF2 overexpression suggests a role in tumor maintenance rather than initiation, which would rather be triggered by other actors, such as WNT pathway. This two-step model is supported by the fact that IGF2 overexpression is found only in ACCs, whereas mutations in β-catenin are reported in both ACAs and ACCs. To test the hypothesis of cooperation between IGF2 and WNT pathways, we generated a mouse model presenting both genetic alterations, by mating ΔCat mice with IGF2 overexpressing mice (28) (Table 1). Our analysis of this model clearly showed that IGF2 overexpression in the context of constitutive WNT/β-catenin pathway activation only had a moderate effect on tumor progression. In another approach, Hammer and collaborators generated a mouse model with both loss of *APC*, to achieve WNT activation, and loss of imprinting at the *Igf2/H19* region, to achieve elevated *IGF2* expression (26) (Table 1). These mice displayed adrenocortical hyperplasia, microscopic and macroscopic adenomas, and cancer formation. Although the phenotype was more severe than that observed in mice presenting loss of APC alone, only one cancer formation was observed. Taken together, these results have clearly shown that genetic alterations in WNT pathway and IGF2 overexpression are not sufficient to trigger malignant adrenocortical tumorigenesis. Interestingly, we recently reported that expression of enhancer of zeste 2 (EZH2), a histone methyl transferase of the polycomb repressive complex 2 (PRC2), positively correlated with malignancy and poor prognosis in three different cohorts of patients with ACTs (102). In this study, we provided evidence that in the H295R human adrenal cancer cell line, EZH2 downregulation or pharmacological inhibition significantly decreased cell proliferation and aggressive behavior and induced apoptosis. Interestingly, EZH2 overexpression was shown to be the result of P53/RB/E2F pathway deregulation in good agreement with ACC omic studies. Although this remains to be demonstrated in a relevant animal model, these data suggest that EZH2 could be involved in malignant progression. Mouse models with various ability to express Ezh2 (loss- and gain-of-function) are, thus, required to decipher the molecular mechanisms involved in EZH2-mediated malignant progression, i.e., transcription repression through PRC2 recruitment or activation (103) and to identify the actors that cooperate with WNT pathway in this process. Altogether, these mouse models indicate that malignant tumors may arise from typical adenomas even if this progression only affects a small proportion of benign tumors and requires additional alterations, such as EZH2 overexpression. One challenge of these studies is the evaluation of tumor staging in mouse models. Until now, mouse tumors have been evaluated with the same criteria as human tumors, i.e., essentially Weiss’s scoring. However, the transposability of these histologic criteria is unclear and the only undoubtful proof of malignant progression is metastatic dissemination, which was not observed in any of the models discussed above. Irrespective of this issue, inheritable tumors in mouse genetic models provide an invaluable tool to follow tumor initiation and progression. Whole genomic analyses in such models would provide a unique opportunity to demonstrate or invalidate the existence of a normal adrenal adenoma–carcinoma continuum in adrenocortical tumorigenesis.

### Pediatric ACCs: TP53, SF1, and Related Mouse Models

In addition to overexpression of IGF2 and activation of WNT pathway, the third most frequent genetic alteration in ACCs is inactivation of TP53/RB pathway. Germline mutations in the *p53* tumor suppressor gene are associated with the development of Li–Fraumeni syndrome (LFS), an autosomal-dominant cancer syndrome resulting in multiple malignancies, including ACCs (104, 105). The rate of germline TP53 mutations in ACCs is age-dependent, ranging from up to 80% in pediatric ACCs (97) to 3–7% in adults (96). A 10-fold increased incidence of ACCs is observed in Southern Brazil due to a germline mutation within the oligomerization domain of *p53* (p.R337H). This germline mutation was first identified in 98% of children with ACCs (106). The p.R337H mutation was also present in 78% of children with sporadic ACTs in another series and in 13% of adult patients with ACCs (107). In adult ACTs, *TP53* mutations are mostly somatic and were considered to represent a later step in tumorigenesis (108, 109). Several mouse models of p53 dysfunction have been generated, including targeted mutations, replicating proteins identified in humans with LFS (110, 111). These mice developed a large spectrum of tumors but not ACCs. Mouse models of adrenal-specific *TP53* loss have not been generated to date, despite the high prevalence of *TP53* mutations in ACCs. However, the adrenocortical dysplasia (*Acd*) mouse model (112), carrying an inactivating mutation in *Tpp/Acd*, which normally functions to protect telomerase, was used to explore the consequences of *TP53* loss during adrenal tumorigenesis (Table 1). Interestingly, ablation of *p53* (obtained by mating *Acd* mice with *p53* null mice) rescued a number of characteristics of the *Acd* phenotype, including adrenal hypoplasia. This indicates that hypoplasia in *Acd* mice results from p53-mediated senescence. Moreover, the loss of *p53* in *Acd* mice leads to development of ACC, suggesting that p53-mediated escape from senescence may contribute to adrenocortical carcinogenesis (29). Another approach to explore the consequences of p53 ablation during adrenal tumorigenesis consists in adrenal targeting of the Simian Virus 40 (SV40) large T antigen (TAg), a potent oncogene acting in part by inactivating p53 [reviewed in (113)]. TAg expressed under the control of several promoters has been used to induce ACTs in transgenic mice. In the *inhibin-*α (*inha*) promoter-TAg mouse, ACCs are induced by gonadectomy (30) (Table 1). This mouse model will not be discussed in details here as mouse models of gonadectomy-induced ACTs are beyond the scope of this review, being unrelated to the context of human adrenal tumors. In order to better recapitulate the context of human adrenal tumor development, we have generated a model in which TAg is expressed under the control of the adrenal cortex specific *Akr1b7* promoter (31) (Table 1). In this AdTAg model 2 of 3 surviving founder mice developed adrenal tumors that were used to generate cell lines for further *in vitro* analysis (114). As exome sequencing and SNP array recently confirmed that p53/RB is one of the most frequently altered pathways in ACCs (11), future mouse models are needed to further explore the role of TP53 in adrenal tumor formation and progression. Considering the well-characterized antagonism of large TAg toward p53 and Rb tumor suppressors, one would predict that the AdTAg model could provide one of the most relevant and simple models to explore adrenal tumorigenic processes.

Transcriptional profiling has demonstrated distinct signatures of adult and pediatric ACCs with the pediatric tumor transcriptome displaying similarities to that of fetal adrenal tissue (115). Moreover, pediatric and adult ACCs differ in several clinical, pathological, and molecular aspects [reviewed in (90)], suggesting that they may represent genetically distinct entities. In addition to *TP53*, the genetic locus containing steroidogenic factor 1 gene (*SF-1*) is commonly amplified and overexpressed in pediatric ACCs (116, 117). SF-1 is a nuclear receptor transcription factor that plays an important role in the regulation of steroidogenic genes, in development and function of the adrenal cortex, and in male sexual differentiation (118, 119). In contrast to children, the frequency of amplification and overexpression in adult patients is very low (120), but it is associated with poor outcome (121). A transgenic mouse model, harboring multiple copies of a yeast artificial chromosome, including the *SF-1* genetic locus, termed YACTR mice, has been generated (32) (Table 1). Although these mice developed adrenocortical hyperplasia that further progressed to adrenal tumors in a dose-dependent manner, the morphology of adrenal tumors differed from human ACTs and displayed a gonadal phenotype that was reminiscent of ACTs occurring in gonadectomized mice. These tumors are thought to arise from pluripotent adreno-gonadal precursor cells lying beneath the outer adrenal connective capsule, which have the potential to differentiate into cells of gonadal phenotype (122). Similarly, in YACTR mice, high levels of *SF-1* may trigger proliferation of pluripotent cells and the development of adrenal tumors with a gonadal phenotype. Despite the differences in tumor phenotypes in human and mice, SF-1 appears to play an important role in adrenal tumorigenesis in both species. Recently, it has been proposed that SF-1 overexpression induces alterations of redox mechanisms, which may contribute to adrenal tumorigenesis. Indeed, inactivation of SF1 target gene *Vnn1*, encoding the Vanin-1 protein involved in the response to oxidative stress, was found to antagonize the development of adrenocortical neoplasia in *SF-1* transgenic mice (123).

### Novel Identified Genes in ACCs: Interplays with WNT Pathway and Need for In Vivo Studies

Recent OMIC studies confirmed that the most frequent genetic alterations in ACCs affect the tumor suppressor gene *TP53* and the oncogene *CTNNB1* (each being altered in ~16% of ACCs) (11, 124). In addition to pointing out alterations in already known drivers (*CTNNB1* and *TP53*), these studies identified new genes not previously reported in ACCs. Among these new genes, *ZNRF3* was found to be the most frequently altered (21% of ACCs). *ZNRF3* encodes a protein with E3 ubiquitin ligase activity, supposed to act as a negative regulator of the WNT/β-catenin pathway (125) (Figure 2). ACCs with alterations in *ZNRF3* locus showed activation of β-catenin target genes, but this activation was weaker than in tumors with *CTNNB1* mutations (11). Interestingly, *ZNRF3* and *CTNNB1* alterations were mutually exclusive. Further functional studies and novel mouse models are needed to further explore the role of *ZNRF3* in ACCs. Recurrent mutations have also been found in several known cell cycle regulators, including *CDKN2A, CDK4, RB1*, and *CCNE1*, confirming the notion that release from p53-sensitive checkpoints is a critical step in the process of adrenal tumorigenesis, a notion that previously emerged from analysis of p53 ablation in *Acd* mice.

Recently, mutations have also been found in *PRKAR1A* (126), a gene mutated in PPNAD (as discussed in Section “Primary Pigmented Nodular Adrenocortical Disease and Related Mouse Models”) and much more rarely in ACAs (as discussed in Section “Cortisol-Producing Adenomas: PKA Pathway, PRKACA Mutations, and Lack of Mouse Models”). These recent reports of *PRKAR1A* mutations expand the role of PKA signaling in ACC. If any functional interplay exists between the WNT/β-catenin and the cAMP/PKA pathways in the pathophysiology of adrenal cortex, this is an important point that remains to be clarified *in vivo* (Figure 3). As reviewed in Drelon’s study (127) data from the literature are in favor of such an interplay. This could influence normal adrenal cortex renewal/zonation as well as the pathophysiology of human adrenal tumors. However, whether these pathways cooperate or antagonize each other remains to be determined. This question could be genetically addressed by generating compound transgenic mice based on already available models carrying β-catenin and PKA constitutive activation (Figure 3) (23, 25).

## Future Directions

The understanding of mechanisms of adrenal tumor progression is crucial for the management and treatment of the disease. Indeed, almost half of ACC patients present with metastatic disease and although mitotane alone or in combination with chemotherapy can improve patients’ survival, there is no efficient treatment for advanced disease. There is, thus, a strong need to generate mouse models resembling human pathology to identify actors involved in adrenal carcinogenesis. Especially, mouse models testing the role of newly identified genes in pediatric and adult ACCs are warranted. Moreover, as recent OMIC approaches confirmed that p53/RB is one of the most frequently altered pathways in ACCs (11), the AdTAg mouse model, previously used to generate cell lines for *in vitro* analyses (114), now becomes a relevant model for the study of ACCs.

The study of molecular mechanisms underlying malignant tumor progression represents a crucial step to develop novel specific drugs. The next step is to test their efficacy through *in vitro* and *in vivo* experiments. The only available “*in vivo*” model is the ACC xenograft obtained by subcutaneous injection of the H295R cell line in nude mice (128). Although this model has been extensively used to evaluate new and established drugs, it is important to take its limitations into account. These include deregulated antitumoral response resulting from immunodeficiency and abnormal or deficient microenvironment associated with subcutaneous injection of cells. Therefore, the AdTAg mouse model could be a useful experimental platform to assess *in vivo* the role of newly identified candidates potentially involved in malignant progression, such as EZH2 (102), using genetic or pharmacological approaches. Considering pediatric adrenal cancers, knock-in mouse models reproducing *TP53* p.R337H mutation found in Brazilian families will be a major advance in our understanding of the pathogenesis of these destructive tumors. There is, thus, a strong need for novel mouse models for preclinical studies.

Finally, high throughput next-generation sequencing technologies have allowed major advances in the knowledge of the genetic bases of ACAs. Among the newly identified genes, *ARMC5, KCNJ5*, and *PRKACA* mutations represent the most frequent genetic defects in PBMAH, APAs, and CPAs, respectively (10, 39, 61, 62, 72–74, 79). Mouse models are required to confirm the driving potential of these genes in the initiation and/or progression of ACAs. In conclusion, we expect that the progress in gene editing methods and the recent identification of new recurrently mutated genes, will soon allow the development of novel mouse models capable of faithfully reproducing human adrenal diseases, overcoming the limitations of current models. These models will be useful, on the one hand, to investigate the mechanisms underlying malignant progression of adrenal tumors and, on the other hand, to develop novel therapeutic approaches.

## Author Contributions

FL and AM wrote the manuscript. All authors edited the manuscript.

## Conflict of Interest Statement

The authors declare that the research was conducted in the absence of any commercial or financial relationships that could be construed as a potential conflict of interest.

## Abbreviations

ACA, adrenocortical adenoma; ACC, adrenocortical carcinoma; ACT, adrenocortical tumor; ACTH, adrenocorticotropin hormone; APA, aldosterone-producing adenoma; CPA, cortisol-producing adenoma; CS, Cushing’s syndrome; GIP, gastric inhibitor polypeptide; GPCR, G-protein-coupled receptors; LH/hCG, luteinizing hormone/choriogonaotropin; PA, primary aldosteronism; PBMAH, primary bilateral macronodular adrenal hyperplasia; PKA, protein kinase A; PPNAD, primary pigmented nodular adrenal hyperplasia; SF-1, steroidogenic factor 1.
